# Retraction: *In silico* investigation of novel *Plasmodium Falciparum* glycogen synthase kinase (*pf*GSk3β) inhibitors for the treatment of malaria infection

**DOI:** 10.1371/journal.pone.0351918

**Published:** 2026-06-23

**Authors:** The PLOS One Editors

Following the publication of this article [1], concerns were raised about the suitability of the methodology to address the stated research aim. Specifically, the study aimed to investigate known glycogen synthase kinase-3 beta (GSK3β) inhibitor molecules using *in silico* analyses to assess their potential for inhibiting *Plasmodium falciparum* GSK3β. The GSK3β structure used in the analyses (PDB 3ZDI) is incorrectly identified in [1] as a 3D model of *P. falciparum* GSK3β; however, PDB 3ZDI reports data for the human GSK3β protein, not the *P. falciparum* GSK3β protein.

In editorial follow-up, the corresponding author stated that the PDB structure was chosen based on the study cited as Reference 18 in [1]. The *PLOS One* Editors consulted with a member of the Editorial Board who advised that the reporting of the results and methodology is insufficiently detailed for accurate replication and interpretation of the work, but that the use of the human GSK3β PDB file is a fundamental flaw in the study design such that the analyses do not support the conclusions regarding potential inhibition of the *P. falciparum* enzyme.

In light of the above concerns about the validity of the results and the support for the central conclusions published in [1], the *PLOS One* Editors retract this article.

KFA responded but expressed neither agreement nor disagreement with the editorial decision. SO either did not respond directly or could not be reached.
